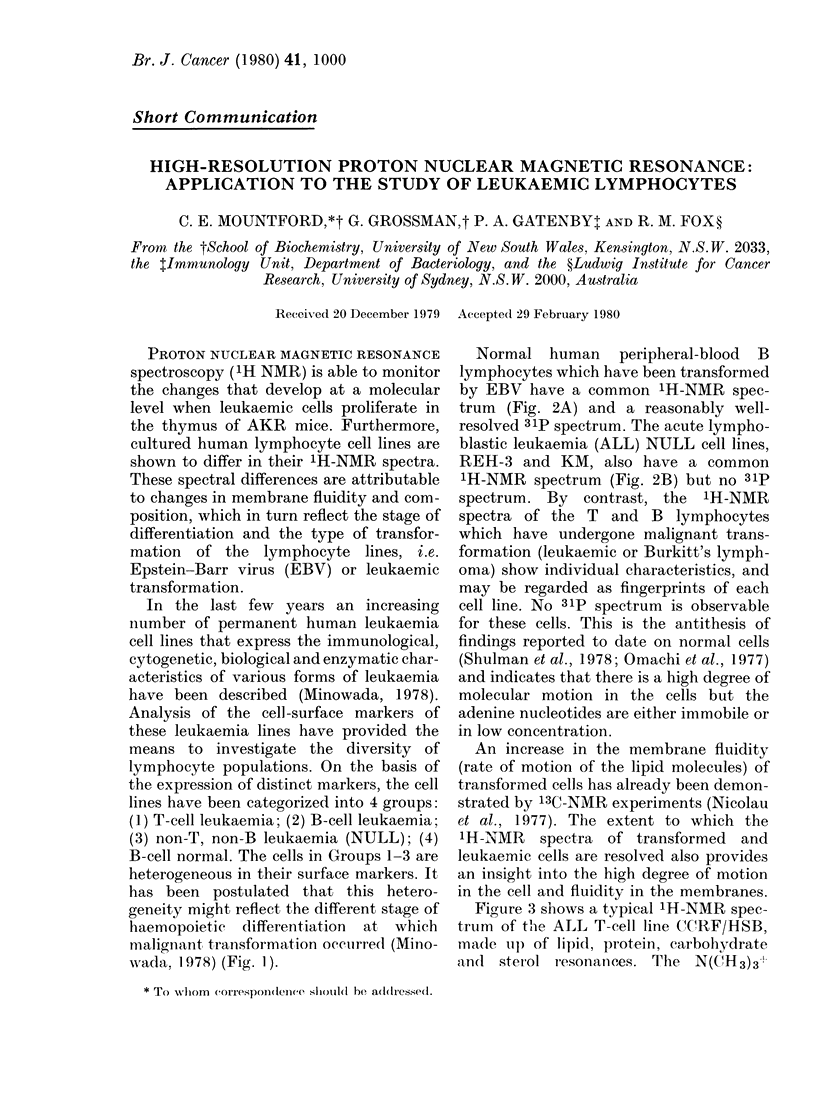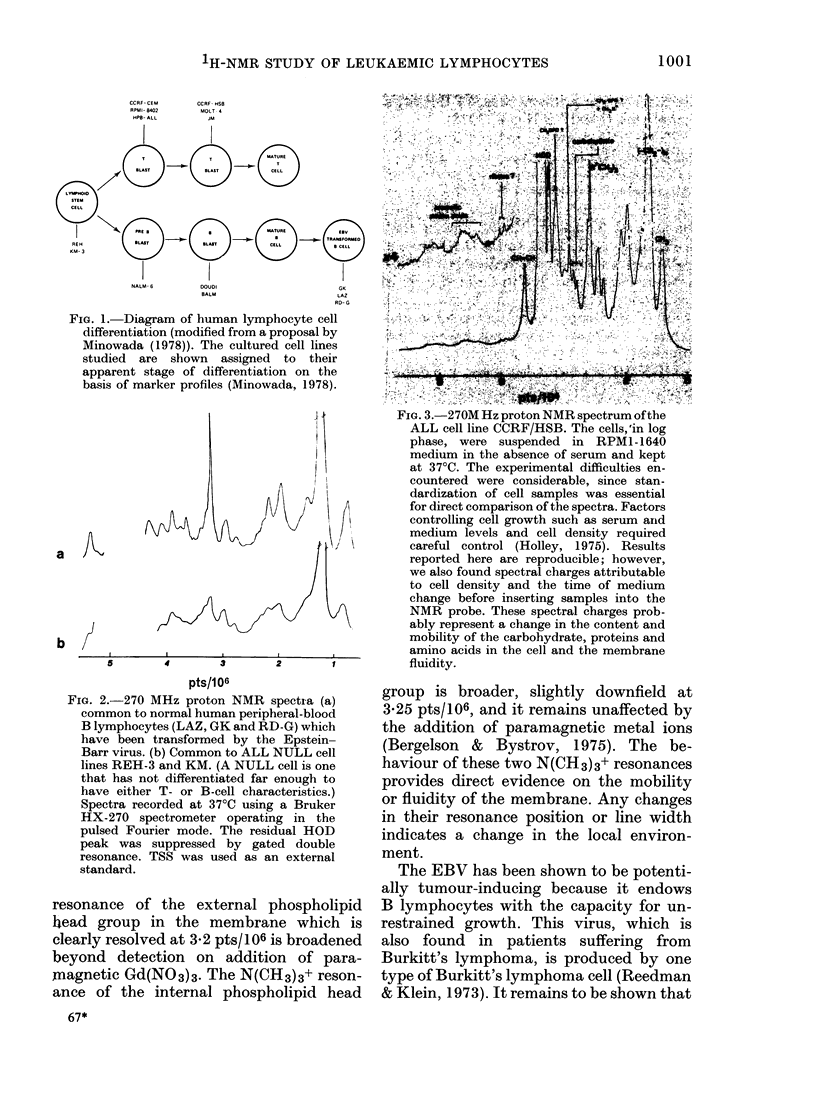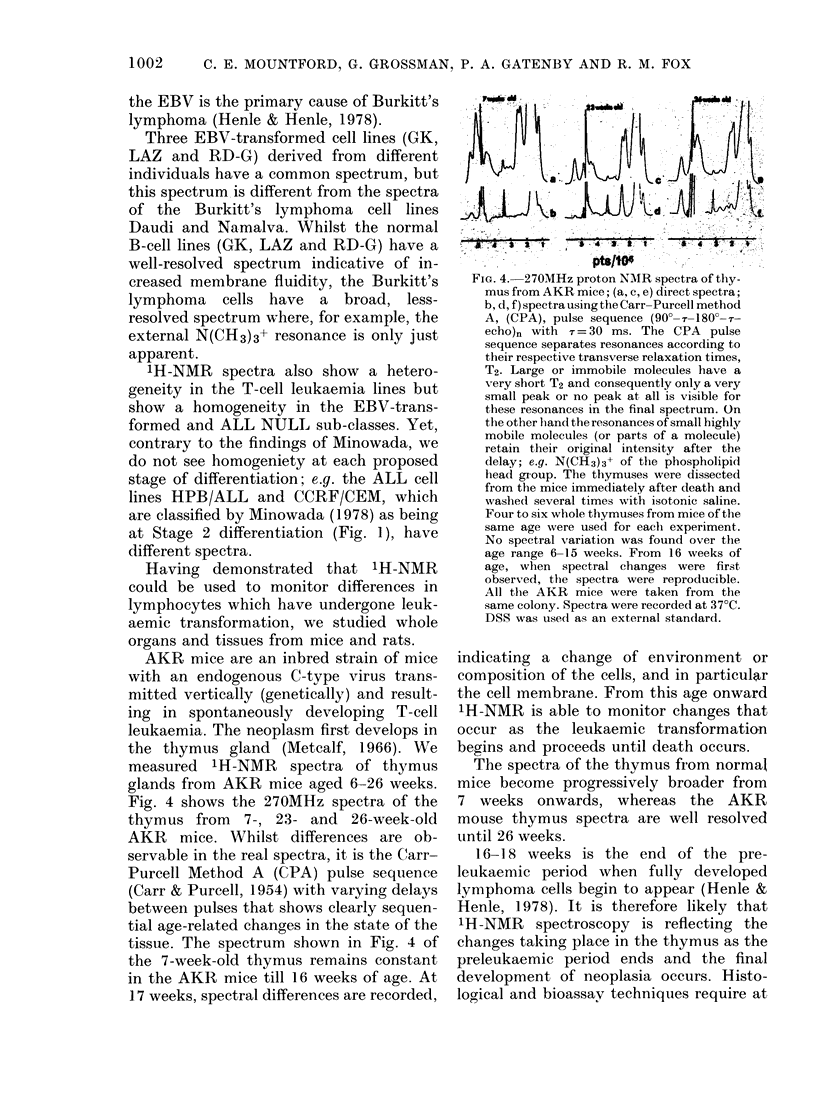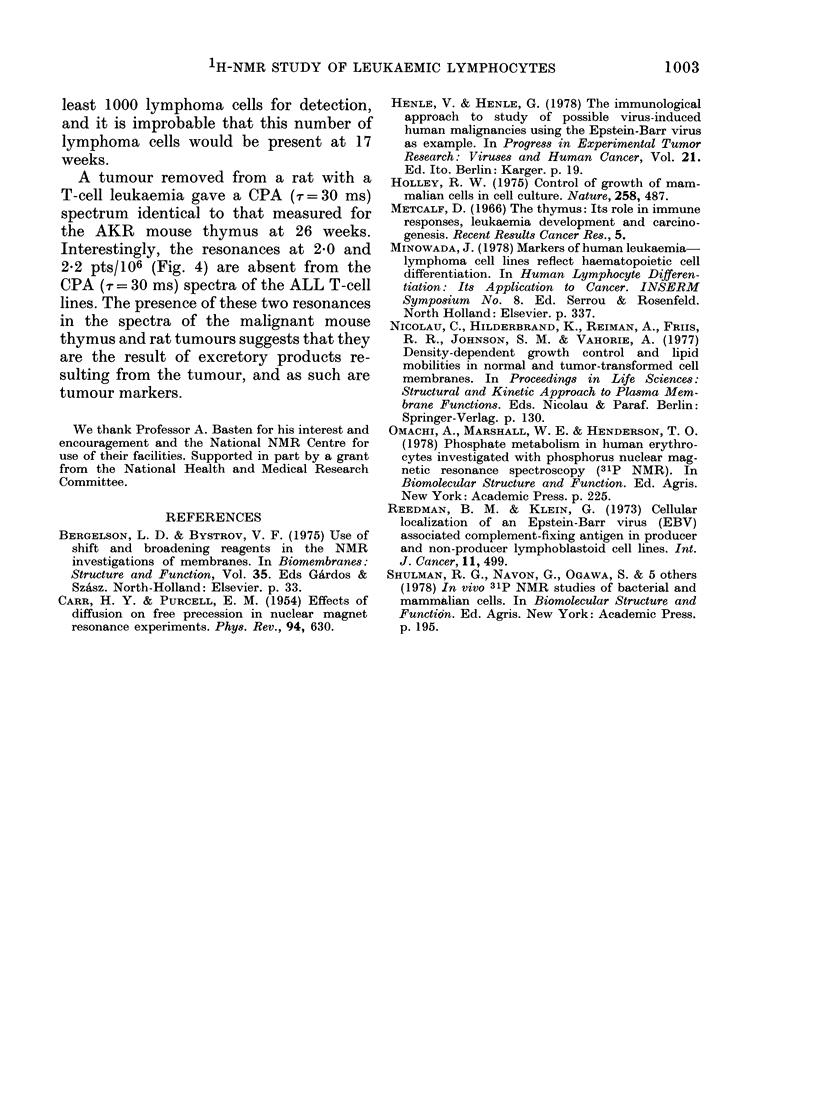# High-resolution proton nuclear magnetic resonance: application to the study of leukaemic lymphocytes.

**DOI:** 10.1038/bjc.1980.182

**Published:** 1980-06

**Authors:** C. E. Mountford, G. Grossman, P. A. Gatenby, R. M. Fox


*Br. J. Cancer* (1980) **41**, 1000

***Short Communication***

### HIGH-RESOLUTION PROTON NUCLEAR MAGNETIC RESONANCE: APPLICATION TO THE STUDY OF LEUKAEMIC LYMPHOCYTES

C. E. MOUNTFORD,*† G. GROSSMAN,† P. A. GATENBY‡ AND R. M. FOX§

*From the †School of Biochemistry, University of New South Wales, Kensington, N.S.W.* 2033, *the ‡Immunology Unit, Department of Bacteriology, and the §Ludwig Institute for Cancer Research, University of Sydney, N.S.W.* 2000, *Australia*

Received 20 December 1979 Accepted 29 February 1980

PROTON NUCLEAR MAGNETIC RESONANCE spectroscopy ($^1$H NMR) is able to monitor the changes that develop at a molecular level when leukaemic cells proliferate in the thymus of AKR mice. Furthermore, cultured human lymphocyte cell lines are shown to differ in their $^1$H-NMR spectra. These spectral differences are attributable to changes in membrane fluidity and composition, which in turn reflect the stage of differentiation and the type of transformation of the lymphocyte lines, *i.e.* Epstein–Barr virus (EBV) or leukaemic transformation.

In the last few years an increasing number of permanent human leukaemia cell lines that express the immunological, cytogenetic, biological and enzymatic characteristics of various forms of leukaemia have been described (Minowada, 1978). Analysis of the cell-surface markers of these leukaemia lines have provided the means to investigate the diversity of lymphocyte populations. On the basis of the expression of distinct markers, the cell lines have been categorized into 4 groups: (1) T-cell leukaemia; (2) B-cell leukaemia; (3) non-T, non-B leukaemia (NULL); (4) B-cell normal. The cells in Groups 1–3 are heterogeneous in their surface markers. It has been postulated that this heterogeneity might reflect the different stage of haemopoietic differentiation at which malignant transformation occurred (Minowada, 1978) (Fig. 1).

Normal human peripheral-blood B lymphocytes which have been transformed by EBV have a common $^1$H-NMR spectrum (Fig. 2A) and a reasonably well-resolved $^{31}$P spectrum. The acute lymphoblastic leukaemia (ALL) NULL cell lines, REH-3 and KM, also have a common $^1$H-NMR spectrum (Fig. 2B) but no $^{31}$P spectrum. By contrast, the $^1$H-NMR spectra of the T and B lymphocytes which have undergone malignant transformation (leukaemic or Burkitt's lymphoma) show individual characteristics, and may be regarded as fingerprints of each cell line. No $^{31}$P spectrum is observable for these cells. This is the antithesis of findings reported to date on normal cells (Shulman *et al.*, 1978; Omachi *et al.*, 1977) and indicates that there is a high degree of molecular motion in the cells but the adenine nucleotides are either immobile or in low concentration.

An increase in the membrane fluidity (rate of motion of the lipid molecules) of transformed cells has already been demonstrated by $^{13}$C-NMR experiments (Nicolau *et al.*, 1977). The extent to which the $^1$H-NMR spectra of transformed and leukaemic cells are resolved also provides an insight into the high degree of motion in the cell and fluidity in the membranes.

Figure 3 shows a typical $^1$H-NMR spectrum of the ALL T-cell line CCRF/HSB, made up of lipid, protein, carbohydrate and sterol resonances. The $N(CH_3)_3^+$

* To whom correspondence should be addressed.

FIG. 1.—Diagram of human lymphocyte cell differentiation (modified from a proposal by Minowada (1978)). The cultured cell lines studied are shown assigned to their apparent stage of differentiation on the basis of marker profiles (Minowada, 1978).

FIG. 2.—270 MHz proton NMR spectra (a) common to normal human peripheral-blood B lymphocytes (LAZ, GK and RD-G) which have been transformed by the Epstein–Barr virus. (b) Common to ALL NULL cell lines REH-3 and KM. (A NULL cell is one that has not differentiated far enough to have either T- or B-cell characteristics.) Spectra recorded at 37°C using a Bruker HX-270 spectrometer operating in the pulsed Fourier mode. The residual HOD peak was suppressed by gated double resonance. TSS was used as an external standard.

FIG. 3.—270M Hz proton NMR spectrum of the ALL cell line CCRF/HSB. The cells, in log phase, were suspended in RPM1-1640 medium in the absence of serum and kept at 37°C. The experimental difficulties encountered were considerable, since standardization of cell samples was essential for direct comparison of the spectra. Factors controlling cell growth such as serum and medium levels and cell density required careful control (Holley, 1975). Results reported here are reproducible; however, we also found spectral charges attributable to cell density and the time of medium change before inserting samples into the NMR probe. These spectral charges probably represent a change in the content and mobility of the carbohydrate, proteins and amino acids in the cell and the membrane fluidity.

resonance of the external phospholipid head group in the membrane which is clearly resolved at 3·2 pts/$10^6$ is broadened beyond detection on addition of paramagnetic $Gd(NO_3)_3$. The $N(CH_3)_3^+$ resonance of the internal phospholipid head group is broader, slightly downfield at 3·25 pts/$10^6$, and it remains unaffected by the addition of paramagnetic metal ions (Bergelson & Bystrov, 1975). The behaviour of these two $N(CH_3)_3^+$ resonances provides direct evidence on the mobility or fluidity of the membrane. Any changes in their resonance position or line width indicates a change in the local environment.

The EBV has been shown to be potentially tumour-inducing because it endows B lymphocytes with the capacity for unrestrained growth. This virus, which is also found in patients suffering from Burkitt's lymphoma, is produced by one type of Burkitt's lymphoma cell (Reedman & Klein, 1973). It remains to be shown that the EBV is the primary cause of Burkitt's lymphoma (Henle & Henle, 1978).

Three EBV-transformed cell lines (GK, LAZ and RD-G) derived from different individuals have a common spectrum, but this spectrum is different from the spectra of the Burkitt's lymphoma cell lines Daudi and Namalva. Whilst the normal B-cell lines (GK, LAZ and RD-G) have a well-resolved spectrum indicative of increased membrane fluidity, the Burkitt's lymphoma cells have a broad, less-resolved spectrum where, for example, the external $N(CH_3)_3^+$ resonance is only just apparent.

$^1$H-NMR spectra also show a heterogeneity in the T-cell leukaemia lines but show a homogeneity in the EBV-transformed and ALL NULL sub-classes. Yet, contrary to the findings of Minowada, we do not see homogeniety at each proposed stage of differentiation; *e.g.* the ALL cell lines HPB/ALL and CCRF/CEM, which are classified by Minowada (1978) as being at Stage 2 differentiation (Fig. 1), have different spectra.

Having demonstrated that $^1$H-NMR could be used to monitor differences in lymphocytes which have undergone leukaemic transformation, we studied whole organs and tissues from mice and rats.

AKR mice are an inbred strain of mice with an endogenous C-type virus transmitted vertically (genetically) and resulting in spontaneously developing T-cell leukaemia. The neoplasm first develops in the thymus gland (Metcalf, 1966). We measured $^1$H-NMR spectra of thymus glands from AKR mice aged 6–26 weeks. Fig. 4 shows the 270MHz spectra of the thymus from 7-, 23- and 26-week-old AKR mice. Whilst differences are observable in the real spectra, it is the Carr–Purcell Method A (CPA) pulse sequence (Carr & Purcell, 1954) with varying delays between pulses that shows clearly sequential age-related changes in the state of the tissue. The spectrum shown in Fig. 4 of the 7-week-old thymus remains constant in the AKR mice till 16 weeks of age. At 17 weeks, spectral differences are recorded, indicating a change of environment or composition of the cells, and in particular the cell membrane. From this age onward $^1$H-NMR is able to monitor changes that occur as the leukaemic transformation begins and proceeds until death occurs.

FIG. 4.—270MHz proton NMR spectra of thymus from AKR mice; (a, c, e) direct spectra; b, d, f) spectra using the Carr–Purcell method A, (CPA), pulse sequence $(90°–\tau–180°–\tau–\text{echo})_n$ with $\tau = 30$ ms. The CPA pulse sequence separates resonances according to their respective transverse relaxation times, $T_2$. Large or immobile molecules have a very short $T_2$ and consequently only a very small peak or no peak at all is visible for these resonances in the final spectrum. On the other hand the resonances of small highly mobile molecules (or parts of a molecule) retain their original intensity after the delay; *e.g.* $N(CH_3)_3^+$ of the phospholipid head group. The thymuses were dissected from the mice immediately after death and washed several times with isotonic saline. Four to six whole thymuses from mice of the same age were used for each experiment. No spectral variation was found over the age range 6–15 weeks. From 16 weeks of age, when spectral changes were first observed, the spectra were reproducible. All the AKR mice were taken from the same colony. Spectra were recorded at 37°C. DSS was used as an external standard.

The spectra of the thymus from normal mice become progressively broader from 7 weeks onwards, whereas the AKR mouse thymus spectra are well resolved until 26 weeks.

16–18 weeks is the end of the preleukaemic period when fully developed lymphoma cells begin to appear (Henle & Henle, 1978). It is therefore likely that $^1$H-NMR spectroscopy is reflecting the changes taking place in the thymus as the preleukaemic period ends and the final development of neoplasia occurs. Histological and bioassay techniques require at least 1000 lymphoma cells for detection, and it is improbable that this number of lymphoma cells would be present at 17 weeks.

A tumour removed from a rat with a T-cell leukaemia gave a CPA ($\tau = 30$ ms) spectrum identical to that measured for the AKR mouse thymus at 26 weeks. Interestingly, the resonances at 2·0 and 2·2 pts/$10^6$ (Fig. 4) are absent from the CPA ($\tau = 30$ ms) spectra of the ALL T-cell lines. The presence of these two resonances in the spectra of the malignant mouse thymus and rat tumours suggests that they are the result of excretory products resulting from the tumour, and as such are tumour markers.

We thank Professor A. Basten for his interest and encouragement and the National NMR Centre for use of their facilities. Supported in part by a grant from the National Health and Medical Research Committee.

### REFERENCES

Bergelson, L. D. & Bystrov, V. F. (1975) Use of shift and broadening reagents in the NMR investigations of membranes. In *Biomembranes: Structure and Function*, Vol. **35**. Eds Gárdos & Szász. North-Holland: Elsevier. p. 33.

Carr, H. Y. & Purcell, E. M. (1954) Effects of diffusion on free precession in nuclear magnet resonance experiments. *Phys. Rev.*, **94**, 630.

Henle, V. & Henle, G. (1978) The immunological approach to study of possible virus-induced human malignancies using the Epstein-Barr virus as example. In *Progress in Experimental Tumor Research: Viruses and Human Cancer*, Vol. **21**. Ed. Ito. Berlin: Karger. p. 19.

Holley, R. W. (1975) Control of growth of mammalian cells in cell culture. *Nature*, **258**, 487.

Metcalf, D. (1966) The thymus: Its role in immune responses, leukaemia development and carcinogenesis. *Recent Results Cancer Res.*, **5**.

Minowada, J. (1978) Markers of human leukaemia—lymphoma cell lines reflect haematopoietic cell differentiation. In *Human Lymphocyte Differentiation: Its Application to Cancer. INSERM Symposium No.* 8. Ed. Serrou & Rosenfeld. North Holland: Elsevier. p. 337.

Nicolau, C., Hilderbrand, K., Reiman, A., Friis, R. R., Johnson, S. M. & Vahorie, A. (1977) Density-dependent growth control and lipid mobilities in normal and tumor-transformed cell membranes. In *Proceedings in Life Sciences: Structural and Kinetic Approach to Plasma Membrane Functions*. Eds. Nicolau & Paraf. Berlin: Springer-Verlag. p. 130.

Omachi, A., Marshall, W. E. & Henderson, T. O. (1978) Phosphate metabolism in human erythrocytes investigated with phosphorus nuclear magnetic resonance spectroscopy ($^{31}$P NMR). In *Biomolecular Structure and Function*. Ed. Agris. New York: Academic Press. p. 225.

Reedman, B. M. & Klein, G. (1973) Cellular localization of an Epstein-Barr virus (EBV) associated complement-fixing antigen in producer and non-producer lymphoblastoid cell lines. *Int. J. Cancer*, **11**, 499.

Shulman, R. G., Navon, G., Ogawa, S. & 5 others (1978) *In vivo* $^{31}$P NMR studies of bacterial and mammalian cells. In *Biomolecular Structure and Function*. Ed. Agris. New York: Academic Press. p. 195.